# A comprehensive characterisation of large-scale expanded human bone marrow and umbilical cord mesenchymal stem cells

**DOI:** 10.1186/s13287-019-1202-4

**Published:** 2019-03-18

**Authors:** Claire Mennan, John Garcia, Sally Roberts, Charlotte Hulme, Karina Wright

**Affiliations:** 1 Institute of Science and Technology in Medicine, Keele University, Keele, Staffordshire, ST4 7QB UK; 2 Robert Jones and Agnes Hunt Orthopaedic Hospital, Oswestry, Shropshire SY20 7AG UK

**Keywords:** Mesenchymal stem cells, Bone marrow, Umbilical cord, Hollow fibre bioreactor, Large-scale expansion, Characterisation, Flow cytometry, Multipotential differentiation, Telomere length

## Abstract

**Background:**

The manufacture of mesenchymal stem/stromal cells (MSCs) for clinical use needs to be cost effective, safe and scaled up. Current methods of expansion on tissue culture plastic are labour-intensive and involve several ‘open’ procedures. We have used the closed Quantum® hollow fibre bioreactor to expand four cultures each of MSCs derived from bone marrow (BM) and, for the first time, umbilical cords (UCs) and assessed extensive characterisation profiles for each, compared to parallel cultures grown on tissue culture plastic.

**Methods:**

Bone marrow aspirate was directly loaded into the Quantum®, and cells were harvested and characterised at passage (P) 0. Bone marrow cells were re-seeded into the Quantum®, harvested and further characterised at P1. UC-MSCs were isolated enzymatically and cultured once on tissue culture plastic, before loading cells into the Quantum®, harvesting and characterising at P1. Quantum®-derived cultures were phenotyped in terms of immunoprofile, tri-lineage differentiation, response to inflammatory stimulus and telomere length, as were parallel cultures expanded on tissue culture plastic.

**Results:**

Bone marrow cell harvests from the Quantum® were 23.1 ± 16.2 × 10^6^ in 14 ± 2 days (P0) and 131 ± 84 × 10^6^ BM-MSCs in 13 ± 1 days (P1), whereas UC-MSC harvests from the Quantum® were 168 ± 52 × 10^6^ UC-MSCs after 7 ± 2 days (P1). Quantum®- and tissue culture plastic-expanded cultures at P1 adhered to criteria for MSCs in terms of cell surface markers, multipotency and plastic adherence, whereas the integrins, CD29, CD49c and CD51/61, were found to be elevated on Quantum®-expanded BM-MSCs. Rapid culture expansion in the Quantum® did not cause shortened telomeres when compared to cultures on tissue culture plastic. Immunomodulatory gene expression was variable between donors but showed that all MSCs upregulated indoleamine 2, 3-dioxygenase (IDO).

**Conclusions:**

The results presented here demonstrate that the Quantum® can be used to expand large numbers of MSCs from bone marrow and umbilical cord tissues for next-generation large-scale manufacturing, without impacting on many of the properties that are characteristic of MSCs or potentially therapeutic. Using the Quantum®, we can obtain multiple MSC doses from a single manufacturing run to treat many patients. Together, our findings support the development of cheaper cell-based treatments.

**Electronic supplementary material:**

The online version of this article (10.1186/s13287-019-1202-4) contains supplementary material, which is available to authorized users.

## Introduction

Bone marrow-derived mesenchymal stem/stromal cells (BM-MSCs) were first described in 1968 [1] and were suggested to have multilineage potential in 1991 [2]. Since then, there has been huge interest in investigating the potential of these cells in the development of cell-based therapies. BM-MSCs have been shown to differentiate into bone, cartilage and fat lineages in vitro [3] and have been used clinically in orthopaedics and in other fields, most notably in the treatment of graft versus host disease (GvHD). Numerous tissue sources for the derivation of MSCs have been identified, including umbilical cord and adipose tissues [4, 5].

The minimal criteria for MSC characterisation were first published in 2006 by the International Society for Cellular Therapy (ISCT) [6], which were subsequently amended for adipose-derived MSCs [7]. In recent years, there has been an increasing body of evidence from in vitro and in vivo studies suggesting that MSCs exert their therapeutic potency via trophic effects on endogenous cells as well as by the secretion of immunomodulatory molecules [8–12]. Further, it is well established that the ability of MSCs to modulate inflammatory processes is enhanced by their stimulation with pro-inflammatory cytokines (present in many disease states), such as tumour necrosis factor-α (TNF-α), interleukin-1β (IL-1β) and interferon-γ (IFN-γ) [9, 13]. A more recent statement paper was published by the ISCT in 2013 [9], which included detailed criteria for the immunomodulatory characteristics of MSCs. This working proposal advises that the characterisation of stem cells for therapy should include activation or ‘licencing’, which involves MSC culture stimulation with IFN-γ, either alone or with the addition of TNF-α [9].

MSCs derived from bone marrow and, more recently, umbilical cords (UC-MSCs) have been receiving increasing interest in the field of clinical orthopaedic regenerative medicine [14–17]. We have previously studied these MSC types separately and have compared the phenotypes of these cells in in vitro studies [5]. We are currently assessing BM-MSCs versus UC-MSCs in pre-clinical in vivo cartilage injury and osteoarthritis models. Autologous culture-expanded BM-MSCs are also being assessed in a randomised clinical trial for cartilage repair, comparing their efficacy versus the current cell therapy ‘gold standard’, autologous chondrocyte implantation (ACI), alone or in combination in the ASCOT trial [18]. BM-MSCs have been used clinically both in autologous [19] and in allogeneic transplantations [20, 21], whereas UC-MSC therapies are being developed as an allogeneic treatment option [22]. Allogeneic cell therapies have many obvious advantages over autologous options as they can be developed as an ‘off-the-shelf’ therapy with one-step treatment and the ability to bank several doses from a single production run, thereby significantly reducing both manufacturing and treatment costs. Moreover, allogeneic cell therapies can be employed when autologous options are not suitable, for instance when autologous donor tissues such as bone marrow might not yield a biologically relevant cell mass, which is likely the case for extremely elderly orthopaedic patients.

There are several publications describing the best practice for the expansion and of the UC-MSC populations using traditional TCP techniques and for the cryogenic banking of UC-MSCs for allogeneic clinical use [23–25]. The good manufacturing practice (GMP) compliant Quantum® bioreactor, manufactured by Terumo BCT, has been used in several pre-clinical and clinical production runs for BM-MSCs and MSCs derived from adipose tissues [26–36], but to our knowledge has not been used to expand UC-MSCs. Sub-populations of cells (distinct in morphology to MSCs) have been described following the initial expansion phase of cells from the bone marrow [37], but these are poorly characterised. The bioreactor itself is made up of ~ 11.5 × 10^3^ polysulphone hollow fibres with semi-permeable membranes, which equates to a surface area of 2 .1 m^2^. Each fibre is approximately 215 μm in diameter and as such is only suitable for thin surface coatings and monolayers of adherent cells, rather than cell clusters and tissues. Monolayer cultures are monitored for growth by analysing media taken from a sample port to measure glucose and lactate concentrations, since these small molecules pass freely across the bioreactor membranes. The system has been shown to effectively scale-up MSC manufacturing, required for the switch from autologous to allogeneic manufacture and multi-dose banking [28, 31]. The proliferative capacity of MSCs at low passage may be critical to forming useful cell banks since there are reports that BM-MSC at higher passage number (passage 5 to 10) may have diminished efficiency and reduced therapeutic effect in vivo [38]; however, no studies to our knowledge have assessed Quantum® products in terms of their longevity at low passage.

In the current study, we have assessed the Quantum® system for the isolation and expansion of BM-MSCs directly from bone marrow aspirate and have re-seeded BM-MSCs into the system for a two-phase Quantum® expansion. We have uniquely and comprehensively characterised BM-MSCs and their sub-populations at both expansion phases. In addition, for the first time to our knowledge, we have assessed the Quantum® bioreactor for the up-scale manufacture of UC-MSC using a ‘hybrid’ process, whereby UC-MSCs have been isolated from tissues onto tissue culture plastic (TCP), as described previously [5, 39], and have been re-seeded into the Quantum® for the second expansion phase. We have aimed to comprehensively characterise the Quantum® BM- and UC-MSC products compared to TCP parallel populations, according to the ISCT criteria for MSCs and using a panel of markers indicative of chondrogenic potency, immunomodulatory capacity, and have assessed their longevity in terms of telomere length.

## Materials and methods

The overall experimental design is illustrated in Additional file 1 to add clarity.

### BM-MSC isolation and expansion

Four BM aspirates were obtained from Lonza (Walkersville, Maryland, WV), sourced from healthy male donors aged 22–32 years, with informed consent. Twenty millilitres of BM was initially loaded into the Quantum® at passage (P) 0. Quantum® expansion methodologies are described in a subsequent section. A parallel culture at P0 was grown on tissue culture TCP for comparison. For TCP cultures, 5 ml of BM was diluted 1:1 with Dulbecco’s phosphate-buffered saline (PBS; Life Technologies, Paisley, UK) and was carefully layered over Lymphoprep (Fresenius Kabi Norge, Norway). Mononuclear cells were isolated after being centrifuged at 900*g* for 20 min, re-suspended in complete medium (containing Dulbecco’s modified Eagle’s medium (DMEM-F12) containing 10% foetal calf serum (FCS; Life Technologies) and 1% penicillin/streptomycin (P/S; Life Technologies)) and centrifuged again at 750*g* for 10 min. The resulting pellet was plated out in a complete medium at a seeding density of 20 × 10^6^ cells per 75-cm^2^ flask. After 24 h, non-adherent cells were removed by changing the medium and adherent cells were cultured in monolayer. A second expansion in the Quantum® (P1) was undertaken after re-seeding the bioreactor with 5–10 × 10^6^ BM-MSCs. Again, a parallel culture of BM-MSCs was grown on TCP for comparison. TCP medium was changed every 2–3 days. All cells were maintained in a humidified atmosphere at 5% CO_2_ and 21% O_2_ at 37 °C until they reached 70–80% confluence at which time cultures were passaged by trypsinisation.

### UC-MSC isolation and expansion

Umbilical cords were collected with informed maternal consent and processed within 24 h of delivery as previously described [5, 39]. Favourable ethical approval was given by the National Research Ethics Service (10/H10130/62). UC-MSCs were obtained by processing ~ 30 cm of whole UC, which was weighed and minced into small pieces (~ 2 mm^3^) before digesting with 1 mg/ml collagenase I (> 125 digesting units/mg; Sigma-Aldrich, Dorset, UK) for 1 h at 37 °C. Tissue was removed from the digest, and the supernatant was centrifuged at 80*g* for 10 min; the pellet was re-suspended in a complete medium (as described for BM-MSCs) and plated onto tissue culture plastic (Sarstedt, Leicester, UK). A ‘hybrid’ process was used for UC-MSC expansion in the Quantum®, whereby UC-MSCs were expanded first on TCP and after the first expansion (P0) 5 × 10^6^ were loaded into the Quantum® system for the second expansion phase (P1). As for BM-MSCs, UC-MSCs were grown in complete media on TCP and in the Quantum®.

### Light microscopy

Phase-contrast images of Quantum®-expanded cells re-seeded onto TCP were taken using the Cell IQ Live Cell Imagining Platform (CM Technologies, Tampere, Finland).

### The Quantum® cell expansion system

The Quantum® system was pre-coated with human cryoprecipitate pooled from five donors (NHS Blood and Transplant, Birmingham, UK) which was diluted 1:1 with PBS. As per the manufacturer’s instructions, to allow for the attachment of the MSCs, the inner surface of the fibres was coated overnight with 100 ml of human cryoprecipitate solution before cell loading. The Quantum® system provides a continuous perfusion of complete medium, whilst simultaneously removing the equivalent volume of conditioned medium. Over the course of an expansion in the Quantum®, the perfusion rate of fresh medium was increased 16-fold to an inlet rate of 1 .6 ml/min, as cell populations grew. Lactate concentration in the conditioned medium was assessed daily using a Lactate Plus meter (Nova Biomedical, Runcorn, UK); this measure was used to inform on incremental increases in media perfusion rate as a surrogate marker of cell number, as per the manufacturer’s instructions.

### Sub-population characterisation in BM-MSC cultures

Cells were harvested by trypsinisation from the Quantum® bioreactor at P0, using 0.25% (*v*/*v*) Trypsin in ethylenediaminetetraacetic acid (EDTA) for 8 min and from sister populations grown on TCP, using 0.05% (*v*/*v*) Trypsin in EDTA for 5 min. Cells were pelleted and re-suspended in 2% bovine serum albumin (BSA) in PBS. Cells were then counted, and 20,000 cells were used for each antibody and the control tube. Cells were stained with the following antibodies, with the purpose of defining macrophage sub-populations and their M1/M2 polarity [40–43]: CD14-Peridinin-chlorophyll proteins-Cy5.5 (PerCP-Cy5.5) (clone M5E2), CD80-Phycoerythrin (PE) (clone L307.4), CD86-Fluorescein isothiocyanate (FITC) (clone FUN-1), CD163-PerCPCy5.5 (clone GH1/61) and CD206-Brilliant violet 421 (BV-421) (clone 19.2). Appropriate isotype-matched IgG controls were used throughout. Cells were analysed on a FACSCanto II flow cytometer using Diva 7 software (Becton Dickinson & company, Oxford, UK). Interleukin-8 (IL-8) in conditioned medium generated from the Quantum® and TCP parallel cultures for freshly isolated BM cultures was assessed using a Quantikine® enzyme-linked immunosorbent assay (R&D Systems, Bio-techne, Minneapolis, MN) according to the manufacturer’s instructions.

### Calculation of growth kinetics

Doubling time (DT) was calculated using the formula DT = (*t*2 − *t*1)ln (2)/ln (*n*2/*n*1) where *n*2 is the cell number at harvesting, *n*1 is the cell number at seeding, *t*2 is the time at cell harvest and *t*1 is the time at seeding. It should be noted that due to the inoculation procedure it is estimated that 30% of the sample is lost in the inoculation loop of the bioreactor at the time of cell seeding.

### Flow cytometry immunoprofiling of MSCs

BM-MSCs and UC-MSCs were harvested by trypsinisation at P0 and P1, pelleted and 20,000 cells per tube were re-suspended in 2% BSA for flow cytometry as described previously. The following antibodies against markers indicative of MSC profiles [6] were used: CD90-PE (clone 5E10), CD105-Allophycocyanin (APC) (clone 266), CD73-BV421 (clone AD2), CD19-BV421 (clone HIB19), CD34-APC (clone 581), CD45-PE (clone HI30), HLA-DR-APC (clone TU36) and CD271-BV421 (clone C40-1457). Integrin profiles were also assessed using antibodies against CD29-APC (clone MAR4), CD49a-PE (clone SR84), CD49b-BV421 (clone 12F1), CD49c-PE (clone C3 II.1) and CD51/61-PE (clone 23C6). Chondrogenic potency markers were assessed using antibodies against CD166-BV421 (clone 3A6), CD39-APC (clone TU66), CD44-PerCp-Cy5.5 (clone G44-26) (all from Becton Dickinson & Company, Oxford, UK), CD151-PE (clone 14A2.H1), receptor tyrosine kinase-like orphan receptor 2 (ROR2)-APC (clone 231509) and fibroblastic growth factor receptor3 (FGFR3)-PE (clone 136334) (R&D systems, Abingdon, UK). Immunomodulatory markers were assessed using the following antibodies: CD40-PE (clone 5C3), CD80-PE (clone L307.4), CD86-FITC (clone 2331), CD106-APC (clone 51-10C9) and CD317-PE (clone 26F8). Appropriate isotype-matched IgG controls were used throughout. Cells were analysed on a FACSCanto II flow cytometer using Diva 7 software (Becton Dickinson & company, Oxford, UK).

### Stimulation of cells with IFN-γ

The pro-inflammatory cytokine IFN-γ (Promokine, Heidelberg, Germany) was used to stimulate cells at a concentration of 25 ng/ml [44]. Following expansion in the Quantum® bioreactor, P1 cells were harvested and seeded onto TCP (P2) and cultured for 24 h at 37 °C, after which IFN-γ was added to their growth media for a further 24 h. RNA was then extracted (as described below), and the expression of several genes was assessed via RT-qPCR.

### Differentiation assays

BM-MSCs and UC-MSCs at P2 were assessed for their osteogenic, adipogenic and chondrogenic differentiation potential using published assays [45, 46]. Cells were seeded at a density of 5 × 10^3^/cm^2^ in 24-well plates (Sarstedt, UK) and grown in monolayer in DMEM F12 and FCS (10%) until they reached ~ 90% confluency, at which time, in test wells (in triplicate), complete medium was replaced with either osteogenic and adipogenic medium. Control wells for each condition were also maintained for 21 days with medium changes every 2–3 days. Osteogenic differentiation medium contained: DMEM F12, FCS (10%), β-glycerophosphate (10 mM), dexamethasone (10 nM) and l-ascorbic-acid (50 μM). Adipogenic differentiation medium contained DMEM F12 and FCS (10%), insulin-transferrin-selenium-X (ITS) (1%) (Gibco, UK), isobutylmethylxanthine (0.5 μM) (Sigma, UK), dexamethasone (1 μM) and indomethacin (100 μM). Osteogenic and adipogenic controls used media containing DMEM F12, FCS (10%). For differentiation assessments at 21 days, cells were fixed with buffered formalin (10%) in PBS for 10 min at room temperature and stained with naphthol-AS-BI phosphate and fast red for 1 h to assess alkaline phosphatase activity for osteogenesis or oil red-O for 1 h to assess lipid formation for adipogenesis.

A pellet culture system was used to assess chondrogenic differentiation potential. Cells (2 × 10^5^) were centrifuged in a 1.5-ml Eppendorf (500*g* for 5 min) in 1 ml of chondrogenic medium consisting of DMEM-F12, FCS (2%), gentamicin (10 μg/ml^−1^), ITS (1%), ascorbic acid (0.1 mM), dexamethasone (10 nM) and transforming growth factor β1 (TGF-β1) (10 ng/ml^−1^) (Peprotech, UK). Pellet cultures were maintained for 21 days, with media changes every 2–3 days. Cell pellets were frozen in liquid nitrogen and stored at − 80 °C prior to use. Pellets were sectioned (7 μM) on a cryostat (Bright Instrument Co Ltd., Huntingdon, UK) onto poly-l-lysine-coated slides and stained for glycosaminoglycans (GAGs) using toluidine blue, a metachromatic stain.

### Toluidine blue staining

Slides with adhered, sectioned pellets were removed from − 20 °C storage and allowed to reach room temperature. Pellets were then flooded with 1% aqueous toluidine blue (BDH) stain solution for 30 s and rinsed in tap water. Slides were left to air dry before mounting under glass coverslips with Pertex mounting medium (Cell Path Ltd).

### RNA isolation and reverse transcriptase-quantitative PCR

RNA was extracted from triplicate cultures of BM-MSC and UC-MSC (P2) in a 24-well plate, with or without exposure to IFN-γ for 24 h. RNA was extracted using the RNeasy Mini kit (Qiagen, Sussex, UK), following the manufacturer’s instructions. RNA was eluted from the spin column with RNAse-free water and stored at − 80 °C until needed. The following genes were assessed: indoleamine 2,3-dioxygenase (IDO), hepatocyte growth factor (HGF), human leukocyte antigen G (HLAG) and prostaglandin E 2 (PGE2). RT-qPCR analysis was performed using the SYBR green mastermix (Applied Biosystems, Warrington, UK) with hypoxanthine phosphoribosyltransferase 1 (HPRT1) and glyceraldehyde 3-phosphate dehydrogenase (GAPDH) as reference genes (Qiagen, QuantiTect Primer Assay).

The reaction was measured in the QuantStudio 3 RT-qPCR system (Applied Biosystems) and C_t_ values using the SDS software (Applied Biosystems). Following normalisation to the reference genes HPRT1 and GAPDH, the presence of genes of interest (mRNA) in cells grown with IFN-γ stimulation was expressed as a ratio compared to those grown in normal media without IFN-γ. For stimulated cells, the gene expression profile of IFN-γ-treated cells was expressed as a ratio compared to un-stimulated cells, using the comparative threshold method [47]. A twofold change threshold (up- or downregulated) was deemed biologically significant.

### Measurement of telomere length

The telomere length for the fastest growing Quantum® product, passage 1 UC-MSCs, was determined and compared to those grown on TCP at the same passage. The reason for assessing telomere length only in UC-MSCs is because of their higher proliferation rate; hence, UC-MSCs are more prone to telomere shortening. DNA was extracted from UC-MSC, grown in the Quantum® bioreactor and on TCP at P1, using the High Pure PCR Template Preparation Kit (Roche, Sussex, UK). Extracted DNA was stored at − 20 °C until needed. Telomere length was determined using the TeloTAGGG kit (Roche, UK) according to the manufacturer’s instructions, from UC-MSC (*n* = 4). One to two microgrammes of genomic DNA from each sample population was digested with a *Hin*fI/*Rsa*I mixture for 2 h at 37 °C and then loaded onto a 0.8% agarose gel. The DNA fragments were separated by gel electrophoresis for 2–4 h at 70 V and transferred to a nylon membrane (Macherey-Nagel, Düren, Germany) by Southern blotting.

The blotted DNA fragments were hybridised to a digoxigenin (DIG)-labelled probe specific for telomeric repeats and incubated with a DIG-specific antibody covalently coupled to alkaline phosphatase, which was visualised by the chemiluminescence substrate CDP-*Star*. The telomere bands were then visualised by exposing the blot to an imaging system (ChemiDoc™ Touch, Bio-Rad, UK) for 5 min. The average terminal restriction fragment (TRF) length was determined for each sample by comparing the signals relative to the molecular weight standard.

### Statistical analysis

Statistical analysis was performed using Prism version 6.0 software (GraphPad Software, La Jolla, CA, USA). A Shapiro-Wilk test for normality was carried out for all datasets, and data were analysed using either parametric or non-parametric tests accordingly. Paired data were analysed using a paired *t* test or a Wilcoxon-matched pairs signed rank test. Data that were unpaired were analysed using an unpaired *t* test or a Mann-Whitney test. *P* values ≤ 0.05 were considered significant.

## Results

### Growth and cell morphology

**Fig. 1:**
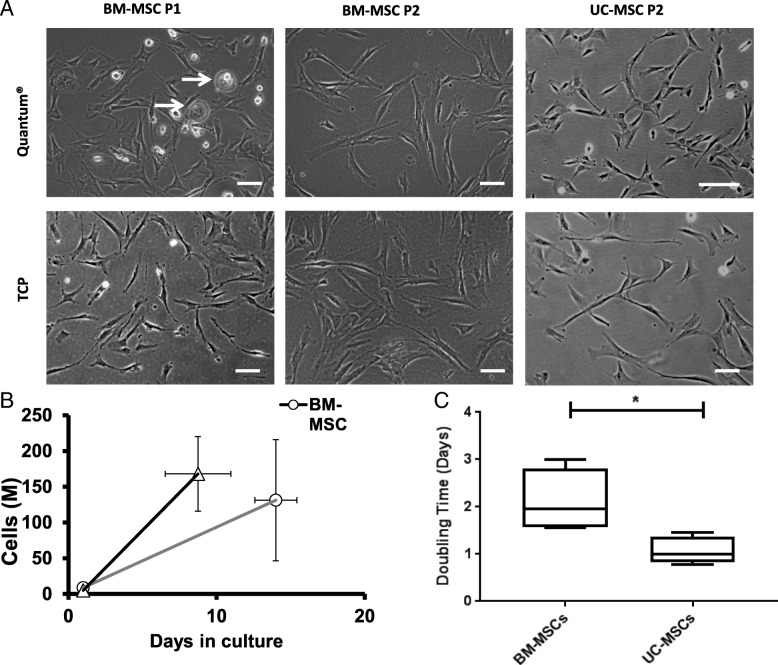
Cell morphologies and growth kinetics of BM- and UC-MSCs expanded in the Quantum® bioreactor and on tissue culture plastic (TCP). **a** Phase-contrast images of BM-MSCs isolated in the Quantum® and re-seeded on TCP at P1 and BM-MSCs and UC-MSCs after P1 expansion in the Quantum®, re-seeded on TCP at P2 (top rows), in comparison to TCP cultures at the same passage (bottom rows) . White arrows indicate macrophage-like cells. Scale bars represent 100 μm. **b** Growth kinetics for BM-MSCs and UC-MSCs grown in the Quantum® at P1. Comparison of MSCs generated from BM (grey line) or UC (black line) in the Quantum at P1 are displayed for comparison. **c** The P1 doubling time of BM-MSCs was significantly longer than UC-MSCs grown in the Quantum®. Mann-Whitney *U* test (*p = 0.029). Mean values are plotted ± standard deviations

BM-MSC cultures isolated and expanded from bone marrow aspirate in the Quantum® and re-seeded onto TCP for imaging showed the presence of typical fibroblast-like MSCs with interspersed morphologically distinct round ‘fried egg’-shaped cells, described by others as indicative of a macrophage or potentially a progenitor sub-population (Fig. 1) [37]. These ‘fried egg’ morphologies were absent in both ‘sister’ populations cultured on TCP and in Quantum® or TCP populations after a second expansion phase, which displayed homogeneous bipolar fibroblastic morphologies (Fig. 1a). Quantum®- and TCP-expanded UC-MSCs at passage 2 showed similar uniform fibroblast-like morphologies (Fig. 1a).

**Fig. 2:**
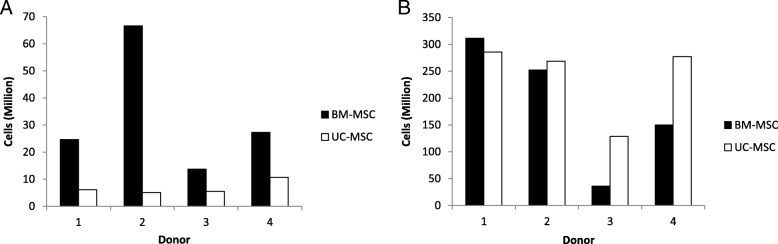
Individual donor BM- and UC-MSC yields at passages 0 and 1. **a** Cell yields from BM-MSC after the first Quantum® harvest (P0) and UC-MSC after harvest from TCP (P0). **b** Cell yields from BM-MSC and UC-MSC after the expansion in the Quantum® at P1

Bone marrow aspirate (20 ml) seeded directly into the Quantum® generated cell yields of 23.1 ± 16.2 × 10^6^ in 14 ± 2 days. In contrast, UCs yielded 6.9 ± 2.6 × 10^6^ UC-MSCs per cord in 17 ± 4 days on TCP (Table 1). At the end of the second expansion, the Quantum® generated a mean cell harvest of 131 ± 84 × 10^6^ BM-MSCs and 168 ± 52 × 10^6^ UC-MSCs. The mean culture time to achieve these numbers for BM-MSCs was 13 ± 1 days and for UC-MSCs was 8 ± 2 days (Fig. 2). Hence, UC-MSC yields were larger and grew significantly faster in the Quantum® bioreactor compared to BM-MSCs (*p = 0.029) (Fig. 1b, c).

**Table 1:** Passage 0 and passage 1 cell growth characteristics of BM- and UC-derived cells in the Quantum® and on TCP

	Donor	1	2	3	4
Passage 0					
BM Quantum®	MNC count (× 10^6^/ml)	30.1	29.9	18.7	21.2
	Cell harvest (× 10^6^)	24.6	66.6	13.7	27.3
	Days in culture	13	13	16	15
UC TCP	Tissue digested (g)	27	25	30	36
	Cell harvest (× 10^6^)	6.1	5.1	5.5	10.7
	Days in culture	20	20	17	12
Passage 1					
BM Quantum®	Cells seeded (× 10^6^)	10	10	5	10
	Cells harvested (× 10^6^)	311.4	252.3	36	150
	Days in culture	12	12	13	15
	Doubling time (days)	1.6	1.6	3.0	2.3
UC Quantum®	Cells seeded (× 10^6^)	5	4.8	5	5
	Cells harvested (× 10^6^)	285.7	268.6	128.6	277.1
	Days in culture	6	11	7	7
	Doubling time (days)	0.8	1.5	1.1	0.9

### BM-MSC sub-population characterisation

**Fig. 3:**
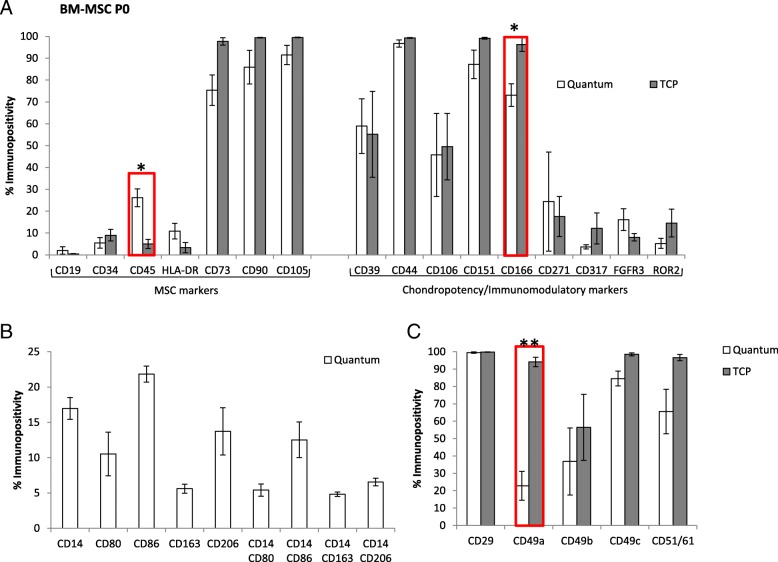
**a**–**c** Characterisation using flow cytometry on Quantum®- and TCP-expanded BM-MSCs at P0. CD45 immunopositivity was significantly greater on cells grown in the Quantum®, but CD166 and CD49a immunopositivity was significantly lower. Paired *t* tests (*p = 0.033, *p = 0.024 and **p = 0.002, respectively). Mean values are plotted ± standard deviations

Bone marrow cultures isolated and grown in the Quantum® (P0) were significantly more immunopositive for CD45 compared to sister populations on TCP (*p = 0.032) (Fig. 3a). In co-expression analyses, we have shown that those cells that displayed positivity for CD14 were also immunopositive for CD80/86 and CD163/206 to varying levels (Fig. 3b). Interleukin-8 secretion into the conditioned media was only detectable in bone marrow cultures isolated and cultured in the Quantum® at P0 and was not detected in the P1 BM-MSC expansion in the bioreactor; this data is included as supplementary material (Additional file 2).

### Immunoprofiling

In bone marrow cultures at P0, the cell adhesion markers CD166 (activated leukocyte cell adhesion molecule) and CD49a (α1 integrin) were both significantly lower on Quantum®-derived cells compared to TCP cultures (*p = 0.024 and **p = 0.002, respectively) (Fig. 3a, c). BM-MSCs grown in the Quantum® and on TCP at passage 0 showed similar immunopositivity for all of the other cell surface markers tested, which are indicative of MSCs, chondrogenic potency and immunomodulatory capacity (Fig. 3a, b).

**Fig. 4:**
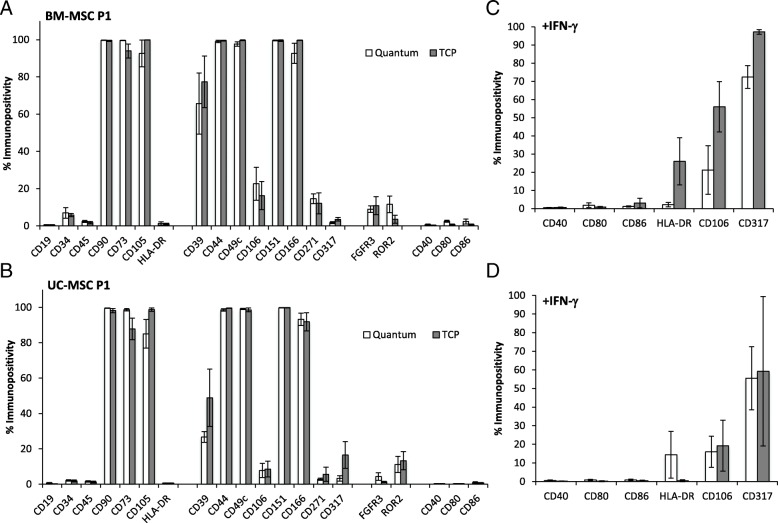
Characterisation using flow cytometry on Quantum®- and TCP-expanded BM-MSCs and UC-MSCs at P1. No significant differences in immunoprofile were noted between culture conditions; however, BM-MSCs (**a**) expressed significantly more CD271 and CD34 compared to UC-MSCs (**b**) after P1 expansion in the Quantum® (*p* = 0.029). Cell surface marker immunopositivity following IFN-ϒ stimulation on P1 Quantum®- and TCP-expanded **c** BM-MSC and **d** UC-MSC re-seeded onto TCP at P2 was comparable for the co-stimulatory markers CD40, CD80 and CD86. Some BM-MSCs grown on TCP and UC-MSCs grown in the Quantum® at P1 and stimulated with IFN-γ at P2 were immunopositive for HLA-DR. All of the cultures tested upregulated CD106 and CD317 in response to IFN-γ, with no significant difference between the Quantum®- and TCP-expanded cells. Mean values are plotted ± standard deviations

At passage 1, no significant differences were observed in marker immunopositivity, when comparing the two conditions or cell types with the exception of two markers, CD271 and CD34. Quantum®-grown BM-MSCs were significantly more immunopositive for CD271 compared to UC-MSCs (14.6 ± 2.6% and 2.8 ± 0.6% respectively, *p = 0.029) and TCP BM-MSCs were more positive for CD34 compared to UC-MSCs (5.9 ± 0.8% and 3.1 ± 0.6% respectively, *p = 0.029) (Fig. 4a, b). IFN-γ stimulation of BM-MSCs and UC-MSCs grown in both conditions did not induce the production of co-stimulatory molecules (CD40, CD80 or CD86) (Fig. 4c, d). HLA-DR was detectable at high levels only in two BM-MSC donors grown on TCP (donor 1 and donor 4, which showed levels of 53.7% and 42.3% on TCP compared to levels of 0.5% and 0.1%, respectively, in the Quantum®) and one UC-MSC donor (donor 2 when grown in the Quantum® showed 52.1% immunopositivity for HLA-DR compared to 0.4% on TCP) (Fig. 4c, d). However, un-stimulated BM and UC populations did not produce HLA-DR. The immunomodulatory markers CD106 and CD317 were also both produced to a higher level on both cell populations following IFN-γ stimulation compared to cells without stimulation; however, there was no significant difference in production between cells grown in the Quantum® or on TCP (Fig. 4c, d).

### Tri-lineage differentiation

**Fig. 5:**
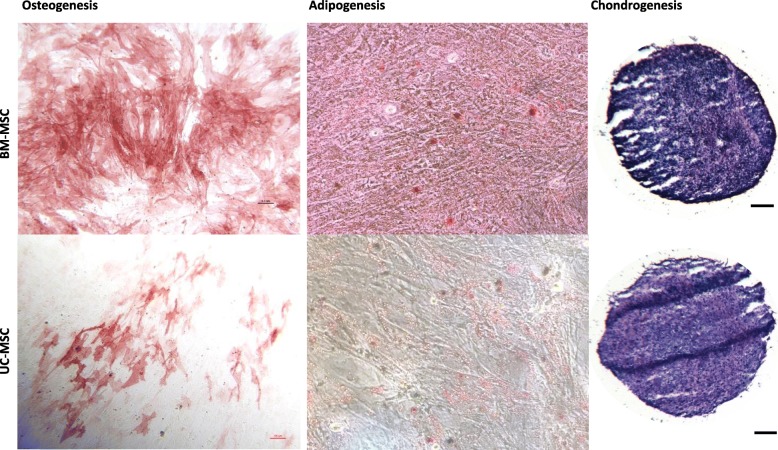
Tri-lineage assessment of the Quantum®-expanded BM- and UC-MSCs. Representative images of osteogenic, adipogenic and chondrogenic differentiation of BM-MSCs (top row) and UC-MSCs (bottom row) grown in the Quantum® bioreactor at P1 and differentiated at P2 for 21 days. BM-MSCs displayed more alkaline phosphatase activity (osteogenesis indicator) and the presence of more lipids (indicative of adipogenesis) compared to UC-MSCs, whereas chondrogenic GAG assessments were comparable across cell types. Scale bars represent 100 μm

The definitive demonstration of multipotency for a cell is the ability to differentiate towards more than one cell type. BM-MSCs and UC-MSCs cultured in the Quantum® at passage 1 and induced at passage 2 showed the potential to differentiate as shown by positive osteogenic, adipogenic and chondrogenic staining (Fig. 5). Adipogenesis was seen with positive Oil red-O staining, and osteogenic differentiation was demonstrated with alkaline phosphatase staining. In addition, metachromatic toluidine blue-stained chondrogenic pellets indicated the presence of GAGs within the pellet matrices.

Some differences were seen between BM-MSCs and UC-MSCs in terms of alkaline phosphatase staining (indicative of osteogenesis), with BM-MSCs showing stronger staining (Fig. 5), comparable to the previous work in our group comparing these MSC sources when grown on TCP [5]. Adipogenic staining of lipids was also more evident in BM-MSC cultures whereas chondrogenic staining of GAGs was comparable between both cell types cultured in the Quantum® (Fig. 5).

### Immunomodulatory gene expression

**Fig. 6:**
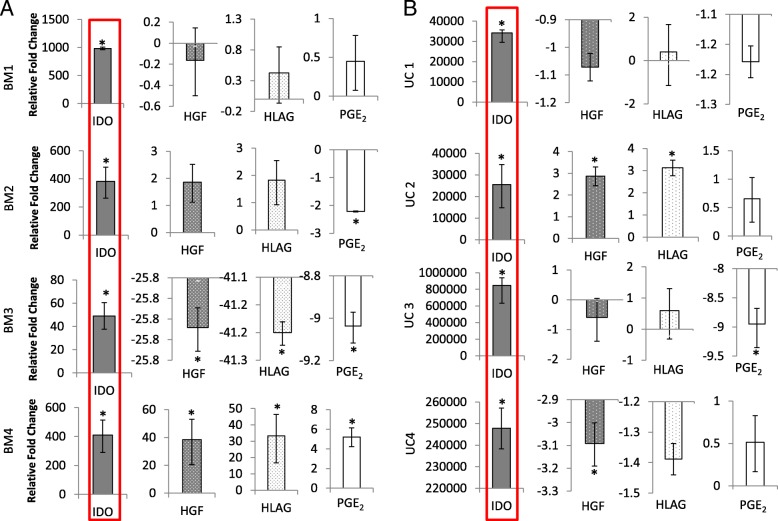
RT-qPCR analyses for the expression of immunomodulatory genes in Quantum®-expanded BM- and UC-MSCs. **a** BM-MSCs and **b** UC-MSCs cultured in the Quantum® bioreactor at passage 2 following stimulation with 25 ng/ml IFN-γ for 24 h. Only IDO was consistently and significantly upregulated in response to IFN-γ stimulation in both cell types. Gene expression was normalised to GAPDH and HPRT1. Gene expression for IFN-γ-stimulated cells is expressed relative to those grown in normal media without the inflammatory stimulus. Stars indicate genes which are significantly up- or downregulated. Mean values are plotted ± standard deviations

Quantum®-derived BM-MSC and UC-MSC cultures at passage 1 showed a consistent and significant upregulation of IDO in response to IFN-γ stimulation in all cultures (Fig. 6). However, the relative fold change observed for IDO in UC-MSCs was much higher than that observed for BM-MSCs. BM3 showed the lowest expression of IDO with a relative fold change of 49, whereas BM 1 showed the highest relative fold change of 984. In contrast, the lowest relative fold change for UCs was UC2 at 25529, with UC3 showing the greatest relative fold change at 847589 (Fig. 6). Variable results for the expression of HGF, HLA-G and PGE_2_ were observed across BM- and UC-MSC populations.

### Analysis of telomere length

**Fig. 7:**
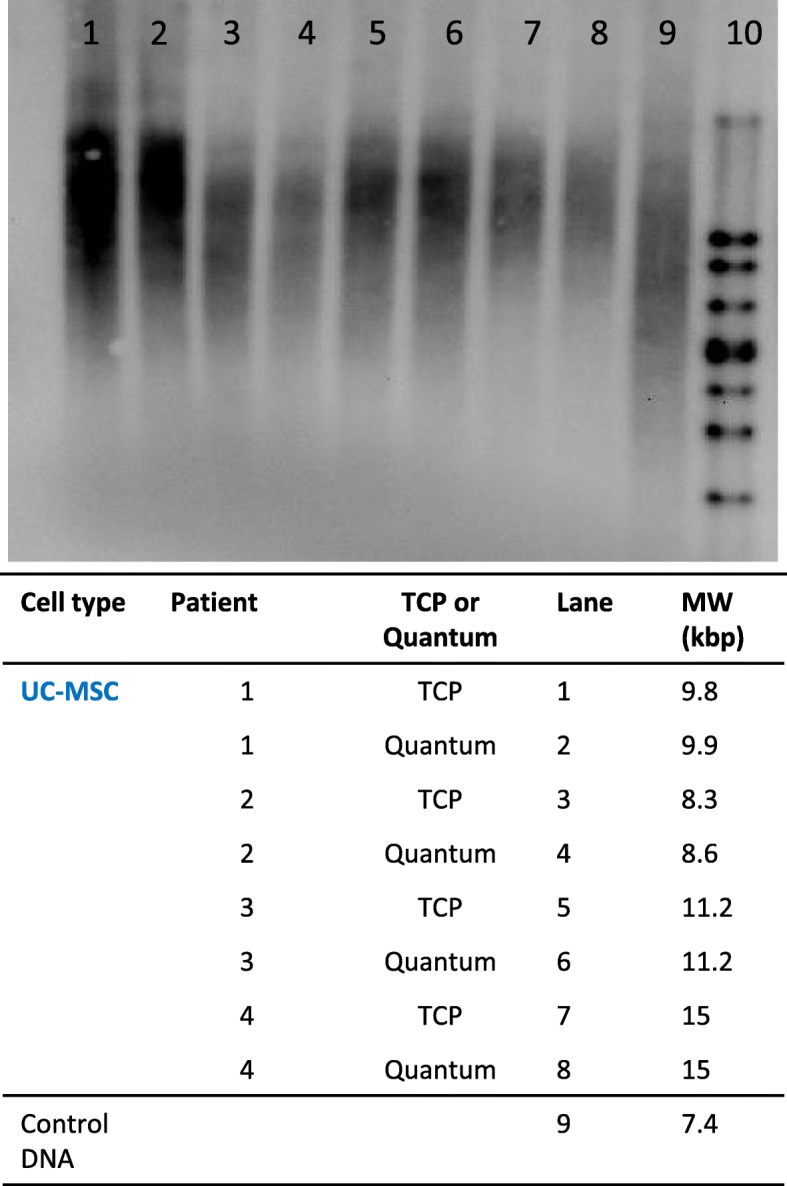
The telomere length of UC-MSCs grown in the Quantum® bioreactor and on tissue culture plastic (TCP) at passage 1. The telomeres of UC-MSC cultures expanded in the Quantum® were the same or greater molecular weight (when assessed via electrophoresis) compared to sister populations expanded on TCP

The rapid expansion of UC-MSCs in the Quantum® did not shorten the telomeres of UC-MSCs compared to those grown on TCP. In fact, UC-MSC 1 and 2 showed slightly longer telomeres when grown in the Quantum® (UC1 9.9 kbp, UC2 8.6 kbp) compared to the same population grown on TCP (UC1 9.8 kbp, UC2 8.3 kbp). UC-MSC 3 and 4 had identical telomere lengths for both culture conditions (Fig. 7).

## Discussion

A strong case exists for the need to develop more cost-effective cell-based therapies. Allogeneic cell expansion using the Quantum® bioreactor clearly has the potential to reduce the cost of producing therapeutic cells ex vivo. Many laboratories have demonstrated the utility of the system for the GMP manufacture of large quantities of MSCs derived from the bone marrow, adipose tissues [26–36] and even neural sources [48]. Quantum® GMP manufacturing protocols have developed from early studies which employed the use of research grade FCS [26, 31], to more recent studies that have switched to pooled human platelet lysate for the supplementation of Quantum® cultures [28, 35, 36]. Similarly, a shift in the preferred choice of substrate utilised to coat the polysulphone hollow fibres inside the bioreactor has been observed, moving from a recombinant fibronectin source to a cryoprecipitate blood product, as a clinical grade source of fibronectin [28, 32, 34]. The innovation described in the current study is the use of the Quantum® system for the expansion of umbilical cord-derived MSCs (UC-MSCs) and the development of a ‘hybrid’ manufacturing protocol for UC-MSC expansion using the Quantum® system. To our knowledge, this has not been demonstrated before in the Quantum® bioreactor. Additionally, we have undertaken a comprehensive phenotypic evaluation of our UC-MSC Quantum® product in terms of ISCT criteria for MSCs, in comparison to sister populations expanded using traditional TCP techniques and BM-MSCs cultured in both ways. A bank of characterisation tests was utilised to study the phenotype further, including a panel of markers indicative of chondrogenic potency and immunomodulatory capacity. In addition, and unlike all other studies using the Quantum® system, we have characterised our UC-MSC Quantum® versus TCP products in terms of telomere length, an important marker linked to cellular ageing [49].

We report that the number of BM- and UC-MSCs generated using the Quantum® system is higher than those expanded using TCP protocols, thus demonstrating an improved potential for the banking of multiple doses of MSCs from both tissue sources for allogeneic treatment. For the first time, we have demonstrated that there are no discernible differences between UC-MSCs grown using up-scale manufacturing techniques or TCP methodologies. Importantly, we have characterised UC-MSCs grown using each methodology in terms of accepted criteria for MSC, which test their response to a pro-inflammatory stimulus, a proposed potential therapeutic mode of action for this cell type. The propagation of large cell doses has important clinical implications for their use in treating indications that might require high cell numbers or for repeat doses. Moreover, it has been suggested that higher cell doses may be required when administering cellular therapies via a systemic route, e.g. intravenous deliveries, compared to a direct application into injured/diseased tissues [50, 51]. Our live cell imaging and flow cytometry data indicated that Quantum®-expanded BM-MSCs and UC-MSCs at passage 1 adhere to the ISCT definition of MSCs and had comparable chondrogenic potency and immunomodulatory immunoprofiles before and after pro-inflammatory stimulation with IFN-γ. This indicates that BM- and UC-MSC populations have retained immunomodulatory phenotypic properties indicative of MSCs [9] following rapid culture expansion in the Quantum® bioreactor. Some donor-donor variability was observed and has been reported previously [39], particularly in terms of HLA-DR upregulation, which highlights the need to screen individual donors rigorously prior to banking for allogeneic use. What we do not yet fully understand are which markers are of importance in terms of potency for cell banking and for which indication, much more work is required to establish such release and banking criteria for clinical MSC products.

Some notable differences in the characteristics of MSCs expanded using the bioreactor compared to TCP are reported in this study. For example, at P0, BM-MSCs grown in the Quantum® showed lower immunopositivity for all of the cell adhesion molecules tested and significantly for CD49a or the integrin α1 subunit. This indicates that there is a differential regulation of adhesion molecules in response to the differing surface chemistries presented and it appears that polystyrene alone cf. polysulfone coated with cryoprecipitate (rich in fibronectin) induces this alteration. Similarly, another study noted the upregulation of CD49a in rat MSCs in response to polystyrene and not on other titanium-coated surfaces [52]. It remains to be seen whether the up/downregulation of adhesion molecules is a beneficial response in terms of the clinical efficacy of these cell populations, but it is likely that some of these molecules are important for the proliferation, attachment and migration of cells through target tissues and matrices [53–55]. In addition, CD49a has been suggested to play an essential role in the regulation of MSC proliferation and cartilage production [56].

Tri-lineage differentiation data demonstrated that whilst UC-MSCs demonstrated comparable chondrogenic and adipogenic differentiation capacity, they showed less alkaline phosphatase staining indicative of poorer osteogenesis. This is not a feature unique to Quantum®-expanded products and is comparable to previously published work in our group where BM-MSCs and UC-MSCs were grown on TCP [5]. However, we acknowledge that alkaline phosphatase staining alone is insufficient to fully determine osteogenic potential and protein assays for typical markers of osteogenesis should be used to further evaluate differentiation status. Whilst these cells have all shown capability to undergo tri-lineage differentiation, the mode of action of MSCs in cartilage repair is still unclear. Rather than MSCs differentiating in vivo to form repair tissues, there is evidence from in vitro and in vivo studies that MSCs exert trophic effects on endogenous cells and secrete immunomodulatory molecules that dampen inflammatory processes contributing to disease progression [8–10].

Our analyses of immunomodulatory genes following IFN-γ stimulation showed variable results between donors, but IDO was consistently upregulated by both BM- and UC-MSCs. The response reported for UC-MSCs was much higher (20–800k relative fold change) compared to BM-MSCs, which expressed a more modest response (50–1000 relative fold change). IDO is a potent immunomodulatory molecule which depletes tryptophan, causing the suppression of T cells [57]. Our group and others have previously demonstrated that TCP-expanded BM- and UC-MSC populations upregulate the IDO gene in response to pro-inflammatory stimulation in vitro, but to varying degrees from different individuals [11, 39]. The reason for the higher expression levels of IDO in UC-MSCs is most likely associated with foetal-maternal tolerance during pregnancy as IDO has an important role to play in protecting the foetus from the maternal immune system [58]. Another study investigating only BM-MSCs reported similar variable upregulation of IDO and suggested that the reason for this was the existence of an intrinsic variation in responsiveness and plasticity of MSC to inflammatory cytokines [11]. This provides additional evidence for the importance of rigorous allogeneic cell bank characterisation and in particular for their immunomodulatory potential tested prior to banking, in order to assure their immunomodulatory function. This is especially important if immunomodulation is the main mechanism of therapeutic action or if cells are being transplanted into inflamed tissues.

Shortened telomeres are known to play an important role in the cellular ageing process [49]. In previous studies, MSCs derived from both bone marrow and umbilical cord tissues have shown that their telomeres shorten progressively during successive cell divisions after ex vivo expansion, down to a threshold length at which time replicative senescence is observed [39, 49]. However, in the current study, no differences in telomere length in Quantum®- and TCP-expanded UC-MSCs (the fastest growing cell population included in the study) were found, which showed that their rapid expansion rate in the bioreactor had not decreased their longevity compared to the same populations grown more slowly on TCP.

Other studies have reported the presence of a sub-population of progenitor cells in Quantum® cultures harvested after bone marrow aspirates were added directly to the bioreactor [59]. The reasons for this sub-population persisting only in Quantum® bone marrow isolations are unclear, but could be related to the lack of bone marrow density gradient centrifugation prior to seeding, or the cryoprecipitate coating of the bioreactor. The mechanism of isolation and the distinct phenotype described for this sub-population warrants further study. We observed a sub-population with a ‘fried egg’-like morphology in primary bone marrow cultures from the bioreactor. Our morphological assessments and the fact that these bone marrow cultures isolated and grown in the Quantum® (passage 0) were significantly more immunopositive for CD45 compared to sister populations on TCP led us to hypothesise that they could represent a macrophage sub-population. We investigated this further, and our co-expression analyses showed that these cells expressed CD14 and were also immunopositive for CD80/86 and CD163/206, indicative of M1 and M2 macrophage polarisation, respectively [60, 61]. Our hypothesis that these cells displayed macrophage-like characteristics was corroborated further by our secretome analysis which only found IL-8 detectable in cultures with this sub-population and not in comparable cultures where this sub-population was absent (in sister populations grown on TCP and following a second round of expansion in the Quantum®). These macrophage-like cells could also have therapeutic applications, especially M2 polarised sub-populations, shown to be present by immunopositivity for CD163/206 and known to possess desirable functions in tissue repair processes [62, 63]. However, we also noted that these macrophage-like cells were lost after a second round of expansion; hence, the Quantum® and TCP techniques produced comparably ‘pure’ MSC populations at the end of P1, which could be important for defining allogeneic cell products and for potential regulatory release criteria.

## Conclusions

When taken together, our results suggest that the semi-automated Quantum® system can be used to rapidly expand large numbers of MSCs from bone marrow and umbilical cord tissues. The Quantum® manufacturing process is less labour-intensive and involves fewer ‘open’ procedures compared to expansion in flasks and as such is likely to be more cost effective, safe and reproducible. The development of an off-the-shelf up-scale manufactured allogeneic cell product could provide great benefit to patients who may not respond to current treatments, for example if their own cells do not grow or do not possess the correct therapeutic phenotype. Further, allogeneic products do not require a harvest procedure and as such the patient will require only one and not two surgical interventions to receive the treatment. In addition, the manufacturing costs of an allogeneic product are likely to be significantly lower and require less time and effort when using the Quantum® compared to traditional TCP expansion techniques [64].

## Additional files

Additional file 1: Schematic of the experimental plan. (TIF 1484 kb)

Additional file 2: Raw data for IL-8 secretome analysis. (DOCX 13 kb)

## Abbreviations

ACI: Autologous chondrocyte implantation
APC: Allophycocyanin
BM-MSCs: Bone marrow-derived mesenchymal stem/stromal cells
BSA: Bovine serum albumin
BV-421: Brilliant violet 421
DIG: Digoxigenin
DMEM: Dulbecco’s modified Eagle’s medium
DT: Doubling time
EDTA: Ethylenediaminetetraacetic acid
FCS: Foetal calf serum
FGFR3: Fibroblastic growth factor receptor3
FITC: Fluorescein isothiocyanate
GAGs: Glycosaminoglycans
GAPDH: Glyceraldehyde 3-phosphate dehydrogenase
GMP: Good manufacturing practice
GvHD: Graft versus host disease
HGF: Hepatocyte growth factor
HLAG: Human leukocyte antigen G
HPRT1: Hypoxanthine phosphoribosyltransferase 1
IDO: Indoleamine 2,3-dioxygenase
IFN-γ: Interferon-γ
IL-1β: Interleukin-1β
IL-8: Interleukin-8
ISCT: International Society for Cellular Therapy
ITS: Insulin-transferrin-selenium-X
P: Passage
P/S: Penicillin/streptomycin
PBS: Phosphate-buffered saline
PE: Phycoerythrin
PerCP-Cy5.5: Peridinin-chlorophyll proteins-Cy5.5
PGE2: Prostaglandin E 2
ROR2: Receptor tyrosine kinase-like orphan receptor 2
TCP: Tissue culture plastic
TGF- β1: Transforming growth factor β1
TNF-α: Tumour necrosis factor-α
TRF: Terminal restriction fragment
UC-MSCs: Umbilical cord-derived mesenchymal stem/stromal cells

## References

[1] Friedenstein AJ, Petrakova KV, Kurolesova AI, Frolova GP (1968). Heterotopic of bone marrow. Analysis of precursor cells for osteogenic and hematopoietic tissues. Transplantation..

[2] Caplan AI (1991). Mesenchymal stem cells. J Orthop Res.

[3] Pittenger MF, Mackay AM, Beck SC, Jaiswal RK, Douglas R, Mosca JD (1999). Multilineage potential of adult human mesenchymal stem cells. Science..

[4] Garcia J, Wright K, Roberts S, Kuiper JH, Mangham C, Richardson J (2016). Characterisation of synovial fluid and infrapatellar fat pad derived mesenchymal stromal cells: the influence of tissue source and inflammatory stimulus. Sci Rep.

[5] Mennan C, Wright K, Bhattacharjee A, Balain B, Richardson J, Roberts S (2013). Isolation and characterisation of mesenchymal stem cells from different regions of the human umbilical cord. Biomed Res Int.

[6] Dominici M, Le Blanc K, Mueller I, Slaper-Cortenbach I, Marini F, Krause D (2006). Minimal criteria for defining multipotent mesenchymal stromal cells. The International Society for Cellular Therapy position statement. Cytotherapy..

[7] Bourin P, Bunnell BA, Casteilla L, Dominici M, Katz AJ, March KL (2013). Stromal cells from the adipose tissue-derived stromal vascular fraction and culture expanded adipose tissue-derived stromal/stem cells: a joint statement of the International Federation for Adipose Therapeutics and Science (IFATS) and the International Society for Cellular Therapy (ISCT). Cytotherapy.

[8] Ryan JM, Barry F, Murphy JM, Mahon BP (2007). Interferon-γ does not break, but promotes the immunosuppressive capacity of adult human mesenchymal stem cells. Clin Exp Immunol.

[9] Krampera M, Galipeau J, Shi Y, Tarte K, Sensebe L (2013). Immunological characterization of multipotent mesenchymal stromal cells--the International Society for Cellular Therapy (ISCT) working proposal. Cytotherapy.

[10] Krampera M, Cosmi L, Angeli R, Pasini A, Liotta F, Andreini A (2006). Role for interferon-gamma in the immunomodulatory activity of human bone marrow mesenchymal stem cells. Stem Cells.

[11] François M, Romieu-Mourez R, Li M, Galipeau J (2012). Human MSC suppression correlates with cytokine induction of indoleamine 2,3-dioxygenase and bystander M2 macrophage differentiation. Mol Ther.

[12] Roelofs AJ, Zupan J, Riemen AHK, Kania K, Ansboro S, White N (2017). Joint morphogenetic cells in the adult synovium. Nat Commun.

[13] Ren G, Su J, Zhang L, Zhao X, Ling W, L’Huillie A (2009). Species variation in the mechanisms of mesenchymal stem cell-mediated immunosuppression. Stem Cells.

[14] Wong KL, Lee KBL, Tai BC, Law P, Lee EH, Hui JHP (2013). Injectable cultured bone marrow–derived mesenchymal stem cells in Varus knees with cartilage defects undergoing high tibial osteotomy: a prospective, randomized controlled clinical trial with 2 years’ follow-up. Arthroscopy.

[15] Chen C, Qu Z, Yin X, Shang C, Ao Q, Gu Y (2016). Efficacy of umbilical cord-derived mesenchymal stem cell-based therapy for osteonecrosis of the femoral head: a three-year follow-up study. Mol Med Rep.

[16] Lamo-Espinosa JM, Mora G, Blanco JF, Granero-Moltó F, Nuñez-Córdoba JM, Sánchez-Echenique C (2016). Intra-articular injection of two different doses of autologous bone marrow mesenchymal stem cells versus hyaluronic acid in the treatment of knee osteoarthritis: multicenter randomized controlled clinical trial (phase I/II). J Transl Med.

[17] Al-Najar M, Khalil H, Al-Ajlouni J, Al-Antary E, Hamdan M, Rahmeh R (2017). Intra-articular injection of expanded autologous bone marrow mesenchymal cells in moderate and severe knee osteoarthritis is safe: a phase I/II study. J Orthop Surg Res.

[18] Richardson JB, Wright KT, Wales J, Kuiper JH, McCarthy HS, Gallacher P (2017). Efficacy and safety of autologous cell therapies for knee cartilage defects (autologous stem cells, chondrocytes or the two): randomized controlled trial design. Regen Med.

[19] Wakitani S, Mitsuoka T, Nakamura N, Toritsuka Y, Nakamura Y, Horibe S (2004). Autologous bone marrow stromal cell transplantation for repair of full-thickness articular cartilage defects in human patellae: two case reports. Cell Transplant.

[20] Gupta PK, Chullikana A, Parakh R, Desai S, Das A, Gottipamula S (2013). A double blind randomized placebo controlled phase I/II study assessing the safety and efficacy of allogeneic bone marrow derived mesenchymal stem cell in critical limb ischemia. J Transl Med.

[21] Vega A, Martín-Ferrero MA, Del Canto F, Alberca M, García V, Munar A (2015). Treatment of knee osteoarthritis with allogeneic bone marrow mesenchymal stem cells: a randomized controlled trial. Transplantation..

[22] Deng D, Zhang P, Guo Y, Lim TO (2017). A randomised double-blind, placebo-controlled trial of allogeneic umbilical cord-derived mesenchymal stem cell for lupus nephritis. Ann Rheum Dis.

[23] Oliver-vila I, Coca MI, Grau-vorster M, Pujals-fonts N, Caminal M, Casamayor-genescà A (2016). Evaluation of a cell-banking strategy for the production of clinical grade mesenchymal stromal cells from Wharton’s jelly. Cytotherapy..

[24] Swamynathan P, Venugopal P, Kannan S, Thej C, Kolkundar U (2014). Are serum-free and xeno-free culture conditions ideal for large scale clinical grade expansion of Wharton’ s jelly derived mesenchymal stem cells ? A comparative study. Stem Cell Res Ther.

[25] Nekanti U, Mohanty L, Venugopal P (2010). Optimization and scale-up of Wharton’ s jelly-derived mesenchymal stem cells for clinical applications. Stem Cell Res.

[26] Jones M, Varella-Garcia M, Skokan M, Bryce S, Schowinsky J, Peters R (2013). Genetic stability of bone marrow-derived human mesenchymal stromal cells in the Quantum System. Cytotherapy..

[27] Nold P, Brendel C, Neubauer A, Bein G, Hackstein H (2013). Good manufacturing practice-compliant animal-free expansion of human bone marrow derived mesenchymal stroma cells in a closed hollow-fiber-based bioreactor. Biochem Biophys Res Commun.

[28] Haack-Sørensen M, Juhl M, Follin B, Harary Søndergaard R, Kirchhoff M, Kastrup J (2018). Development of large-scale manufacturing of adipose-derived stromal cells for clinical applications using bioreactors and human platelet lysate. Scand J Clin Lab Invest.

[29] Rojewski MT, Fekete N, Baila S, Nguyen K, Fürst D, Antwiler D (2013). GMP-compliant isolation and expansion of bone marrow-derived MSCs in the closed, automated device quantum cell expansion system. Cell Transplant.

[30] Hanley PJ, Mei Z, Durett AG, da Graca C-HM, Klis M, Li W (2014). Efficient manufacturing of therapeutic mesenchymal stromal cells with the use of the quantum cell expansion system. Cytotherapy.

[31] Lechanteur C, Baila S, Janssens ME, Giet O, Briquet A, et al. Large-scale clinical expansion of mesenchymal stem cells in the GMP-compliant, closed automated Quantum® cell expansion system: comparison with expansion in traditional T-flasks. J Stem Cell Res Ther. 2014;4. 10.4172/2157-7633.1000222.

[32] Haack-Sørensen M, Follin B, Juhl M, Brorsen SK, Søndergaard RH, Kastrup J (2016). Culture expansion of adipose derived stromal cells. A closed automated quantum cell expansion system compared with manual flask-based culture. J Transl Med.

[33] Lambrechts T, Papantoniou I, Rice B, Schrooten J, Luyten FP, Aerts JM (2016). Large-scale progenitor cell expansion for multiple donors in a monitored hollow fibre bioreactor. Cytotherapy.

[34] Kastrup J, Haack-Sorensen M, Juhl M, Sondergaard R, Follin B, Lund L (2017). Cryopreserved off-the-shelf allogeneic adipose- derived stromal cells for therapy in patients with ischemic heart disease and heart failure — a safety study. Stem Cells Transl Med.

[35] Kozanoglu I, Maytalman E, Gereklioglu C, Yeral M, Buyukkurt N, Aytan P (2017). Quantum cell expansion system: safe and rapid expansion. Cytotherapy.

[36] Pirrone C, Gobbetti A, Caprara C, Bernardini G, Gornati R, Soldati G (2017). Chondrogenic potential of hASCs expanded in flask or in a hollow-fiber bioreactor. J Stem Cell Res Med.

[37] Petrini M, Pacini S, Trombi L, Fazzi R, Montali M, Ikehara S (2009). Identification and purification of mesodermal progenitor cells from human adult bone marrow. Stem Cells Dev.

[38] Crisostomo PR, Wang M, Wairiuko GM, Morrell ED, Terrell AM, Seshadri P (2006). High passage number of stem cells adversely affects stem cell activation and myocardial protection. Shock..

[39] Mennan C, Brown S, McCarthy H, Mavrogonatou E, Kletsas D, Garcia J (2016). Mesenchymal stromal cells derived from whole human umbilical cord exhibit similar properties to those derived from Wharton’s jelly and bone marrow. FEBS Open Bio.

[40] Stelter F (2000). Structure/function relationships of CD14. Chem Immunol.

[41] Rugtveit J, Bakka A, Brandtzaeg P (1997). Differential distribution of B7.1 (CD80) and B7.2 (CD86) costimulatory molecules on mucosal macrophage subsets in human inflammatory bowel disease (IBD). Clin Exp Immunol.

[42] Fabriek BO, Dijkstra CD, van den Berg TK (2005). The macrophage scavenger receptor CD163. Immunobiology..

[43] Gazi U, Martinez-Pomares L (2009). Influence of the mannose receptor in host immune responses. Immunobiology.

[44] Deuse T, Stubbendorff M, Tang-Quan K, Phillips N, Kay MA, Eiermann T (2011). Immunogenicity and immunomodulatory properties of umbilical cord lining mesenchymal stem cells. Cell Transplant.

[45] Pittenger MF, Mackay AM, Beck SC, Jaiswal RK (1999). Multilineage potential of adult human mesenchymal stem cells.

[46] Jaiswal N, Haynesworth SE, Caplan AI, Bruder SP (1997). Osteogenic differentiation of purified, culture-expanded human mesenchymal stem cells in vitro. J Cell Biochem.

[47] Livak KJ, Schmittgen TD (2001). Analysis of relative gene expression data using real-time quantitative PCR and the 2(−Delta Delta C(T)) method. Methods..

[48] Tirughana R, Metz MZ, Li Z, Hall C, Hsu D, Beltzer J (2018). GMP production and scale-up of adherent neural stem cells with a quantum cell expansion system. Mol Ther Methods Clin Dev.

[49] Bonab M, Alimoghaddam K, Talebian F, Ghaffari S, Ghavamzadeh A, Nikbin B (2006). Aging of mesenchymal stem cell in vitro. BMC Cell Biol.

[50] Muller-Ehmsen J (2012). The problem is obvious, the solution is not: numbers do matter in cardiac cell therapy!. Cardiovasc Res.

[51] Kean TJ, Lin P, Caplan AI, Dennis JE (2013). MSCs: delivery routes and engraftment, cell-targeting strategies, and immune modulation. Stem Cells Int.

[52] ter Brugge PJ, Torensma R, De Ruijter JE, Figdor CG, Jansen JA (2002). Modulation of integrin expression on rat bone marrow cells by substrates with different surface characteristics. Tissue Eng.

[53] Pozzi A, Wary KK, Giancotti FG, Gardner HA (1998). Integrin alpha1beta1 mediates a unique collagen-dependent proliferation pathway in vivo. J Cell Biol.

[54] Hou G, Mulholland D, Gronska MA, Bendeck MP (2000). Type VIII collagen stimulates smooth muscle cell migration and matrix metalloproteinase synthesis after arterial injury. Am J Pathol.

[55] Senger DR, Perruzzi CA, Streit M, Koteliansky VE, de Fougerolles AR, Detmar M (2002). The alpha(1)beta(1) and alpha(2)beta(1) integrins provide critical support for vascular endothelial growth factor signaling, endothelial cell migration, and tumor angiogenesis. Am J Pathol.

[56] Ekholm E, Hankenson KD, Uusitalo H, Hiltunen A, Gardner H, Heino J (2002). Diminished callus size and cartilage synthesis in alpha 1 beta 1 integrin-deficient mice during bone fracture healing. Am J Pathol.

[57] Le Rond S, Gonzalez A, Gonzalez ASL, Carosella ED, Rouas-Freiss N (2005). Indoleamine 2,3 dioxygenase and human leucocyte antigen-G inhibit the T-cell alloproliferative response through two independent pathways. Immunology..

[58] Hao K, Zhou Q, Chen W, Jia W, Zheng J, Kang J (2013). Possible role of the ‘IDO-AhR axis’ in maternal-foetal tolerance. Cell Biol Int.

[59] Savelli S, Trombi L, D’Alessandro D, Moscato S, Pacini S, Giannotti S (2018). Pooled human serum: a new culture supplement for bioreactor-based cell therapies. Preliminary results. Cytotherapy.

[60] Mosser DM, Edwards JP (2008). Exploring the full spectrum of macrophage activation. Nat Rev Immunol.

[61] Martinez FO, Gordon S (2014). The M1 and M2 paradigm of macrophage activation: time for reassessment. F1000Prime Rep.

[62] Mantovani A, Biswas SK, Galdiero MR, Sica A, Locati M (2013). Macrophage plasticity and polarization in tissue repair and remodelling. J Pathol.

[63] Fahy N, Farrell E, Ritter T, Ryan AE, Murphy JM (2015). Immune modulation to improve tissue engineering outcomes for cartilage repair in the osteoarthritic joint. Tissue Eng Part B Rev.

[64] Russell AL, Lefavor RC, Zubair AC (2018). Characterization and cost-benefit analysis of automated bioreactor-expanded mesenchymal stem cells for clinical applications. Transfusion..

