# Supplementary figures and images for: A comprehensive characterisation of large-scale expanded human bone marrow and umbilical cord mesenchymal stem cells
Source: Stem Cell Res Ther. 2019 Mar 18;10:99. doi: 10.1186/s13287-019-1202-4 (PMC6421680; doi:10.1186/s13287-019-1202-4)

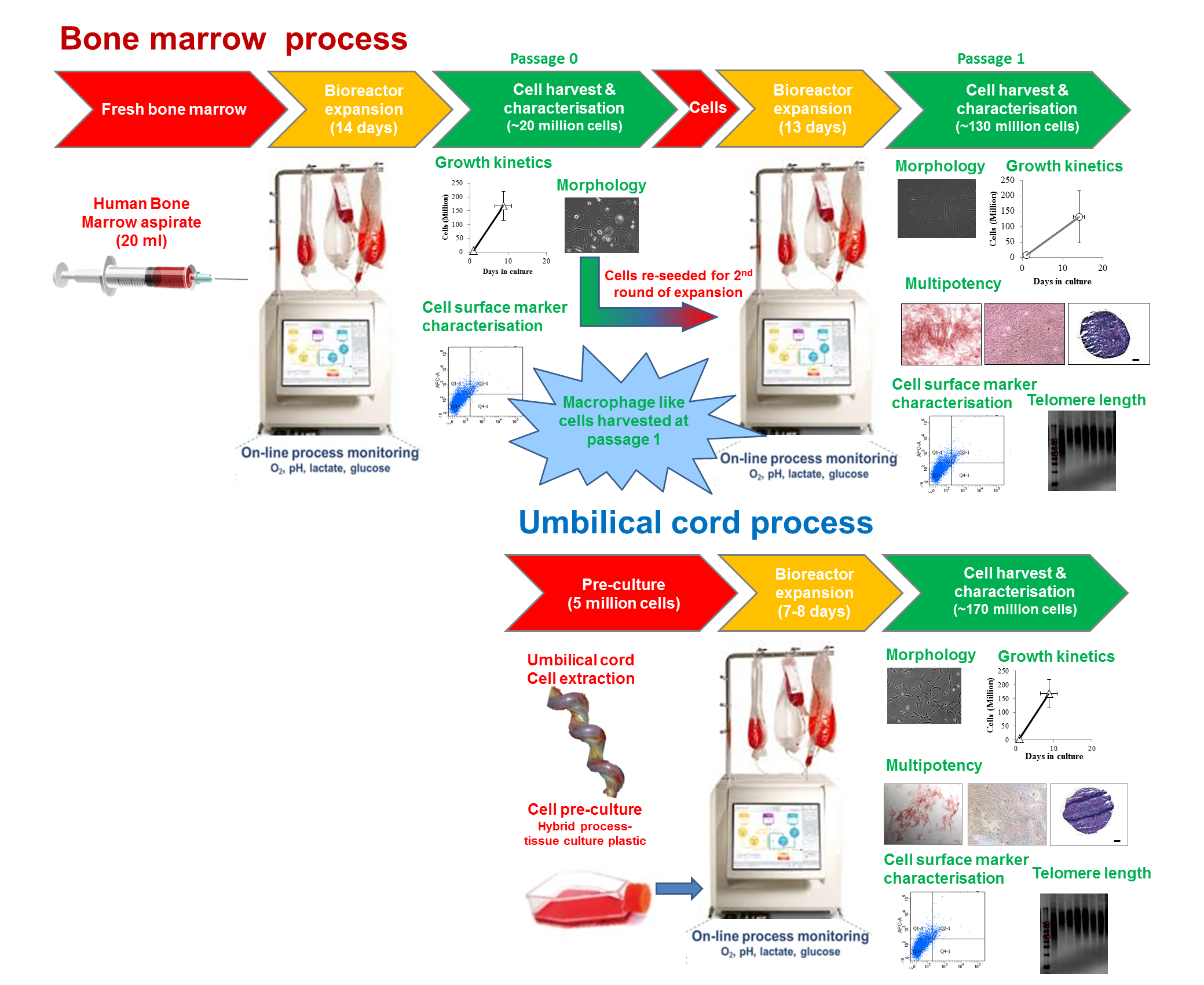

Supplement: Supplementary file 1 — Schematic of the experimental plan. (TIF 1484 kb) [file 13287_2019_1202_MOESM1_ESM.tif]
