# Supplementary material for: A comprehensive characterisation of large-scale expanded human bone marrow and umbilical cord mesenchymal stem cells
Source: Stem Cell Res Ther. 2019 Mar 18;10:99. doi: 10.1186/s13287-019-1202-4 (PMC6421680; doi:10.1186/s13287-019-1202-4)
Supplement: Supplementary file 2 — Raw data for IL-8 secretome analysis. (DOCX 13 kb) [file 13287_2019_1202_MOESM2_ESM.docx]

| IL-8 levels in conditioned medium (pg/ml) | | |
| --- | --- | --- |
| BM-MSCs |  |  |
|  | P1 | P2 |
| Donor 1 | 77.28152 | 0 |
| Donor 2 | 69.85974 | 0 |
| Donor 3 | 65.38376 | 0 |
| Donor 4 | 61.72571 | 0 |
